# (2.2.2-Cryptand)potassium tetra­kis­(η^2^-ethyl­ene)cobaltate(−I)

**DOI:** 10.1107/S1600536812038287

**Published:** 2012-09-12

**Authors:** William W. Brennessel, John E. Ellis

**Affiliations:** a Department of Chemistry, 207 Pleasant Street SE, University of Minnesota, Minneapolis, MN 55455, USA

## Abstract

The title salt, [K(C_18_H_36_N_2_O_6_)][Co(C_2_H_4_)_4_], is one of only two known homoleptic ethyl­enemetalates. The cation and anion are well separated, which gives an unperturbed tetra­hedral anion as is expected for a formally Co^−I^ *d* ^10^ metal center. The considerable elongation of the C=C bonds of the ethyl­ene ligands [average 1.401 (6) Å], relative to that of free ethyl­ene (1.333 Å), is consistent with metal→π* back-bonding models. One arm of the 2.2.2-cryptand (4,7,13,16,21,24-hexa­oxa-1,10-diaza­bicyclo­[8.8.8]hexa­cosa­ne) complexant is disordered and was modeled over two positions with a refined occupancy ratio of 0.559 (2):0.441 (2). In the crystal, the cationic K(2.2.2-cryptand) units are linked *via* C—H⋯O hydrogen bonds, forming inversion dimers. There are no other significant inter­molecular inter­actions in the crystal structure.

## Related literature

For reports on the only other homoleptic ethyl­enemetalate, the ethyl­eneferrate, see: Jonas (1979 ▶, 1981 ▶); Jonas *et al.* (1979 ▶); Jonas & Krüger (1980 ▶). For reports on the anion of the title complex, but with different cations, see: Jonas (1979 ▶, 1981 ▶, 1984 ▶, 1985 ▶); Jonas *et al.* (1979 ▶); Jonas & Krüger (1980 ▶). For the initial report of this anion synthesized from cobalt(II) bromide, see: Brennessel *et al.* (2006 ▶). For neutral and cationic structurally characterized homoleptic ethyl­ene transition metal complexes, see for [Pt^0^]: Howard *et al.* (1983 ▶); for [Cu^+^]: Santiso-Quiñones *et al.* (2007 ▶); for [Ag^+^]: Reisinger *et al.* (2009 ▶); for [Au^+^]: Dias *et al.* (2008 ▶). For details of the preparation and purification of reagents and solvents, and for descriptions of the equipment and techniques, see: Brennessel (2009 ▶). For a description of the Cambridge Structural Database, see: Allen (2002 ▶). For the bond-length of ethyl­ene gas, see: Lide (2003 ▶).
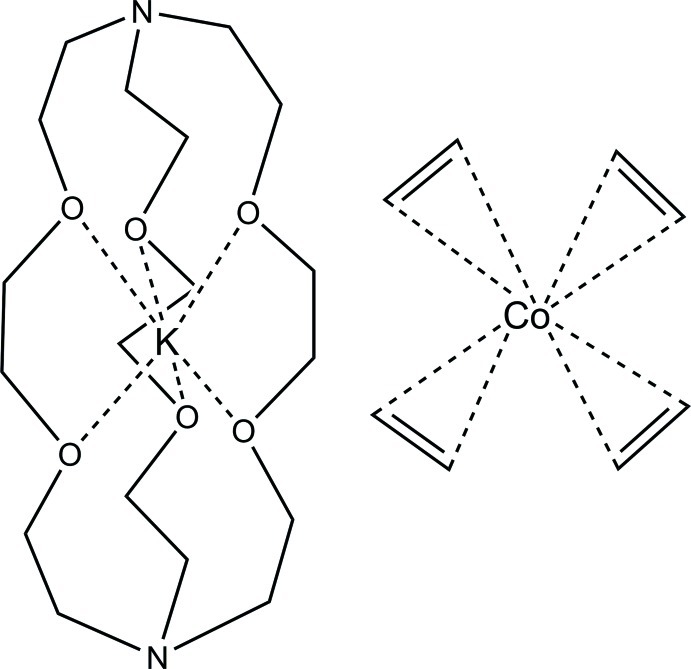


## Experimental

### 

#### Crystal data

[K(C_18_H_36_N_2_O_6_)][Co(C_2_H_4_)_4_]
*M* *_r_* = 586.73
Orthorhombic, 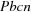

*a* = 25.836 (3) Å
*b* = 10.4820 (12) Å
*c* = 22.544 (3) Å
*V* = 6105.4 (12) Å^3^
*Z* = 8
Mo *K*α radiation
μ = 0.74 mm^−1^
*T* = 173 K
0.50 × 0.24 × 0.16 mm

#### Data collection

Siemens SMART CCD Platform diffractometer
Absorption correction: multi-scan (*SADABS*; Sheldrick, 1996 ▶) *T*_min_ = 0.709, *T*_max_ = 0.891
45000 measured reflections
7010 independent reflections
4825 reflections with *I* > 2σ(*I*)
*R*_int_ = 0.056

#### Refinement

*R*[*F* ^2^ > 2σ(*F* ^2^)] = 0.035
*wR*(*F* ^2^) = 0.073
*S* = 1.02
7010 reflections
414 parameters
16 restraints
H atoms treated by a mixture of independent and constrained refinement
Δρ_max_ = 0.31 e Å^−3^
Δρ_min_ = −0.31 e Å^−3^

### 

Data collection: *SMART* (Bruker, 2003 ▶); cell refinement: *SAINT* (Bruker, 2003 ▶); data reduction: *SAINT*; program(s) used to solve structure: *SIR97* (Altomare *et al.*, 1999 ▶); program(s) used to refine structure: *SHELXL97* (Sheldrick, 2008 ▶); molecular graphics: *SHELXTL* (Sheldrick, 2008 ▶); software used to prepare material for publication: *SHELXTL*.

## Supplementary Material

Crystal structure: contains datablock(s) I, global. DOI: 10.1107/S1600536812038287/su2495sup1.cif

Structure factors: contains datablock(s) I. DOI: 10.1107/S1600536812038287/su2495Isup2.hkl

Additional supplementary materials:  crystallographic information; 3D view; checkCIF report

## Figures and Tables

**Table 1:** Hydrogen-bond geometry (Å, °)

*D*—H⋯*A*	*D*—H	H⋯*A*	*D*⋯*A*	*D*—H⋯*A*
C24—H24*A*⋯O5^i^	0.99	2.59	3.326 (6)	131

Symmetry code: (i) 
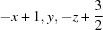
.

**Fig. 1.**
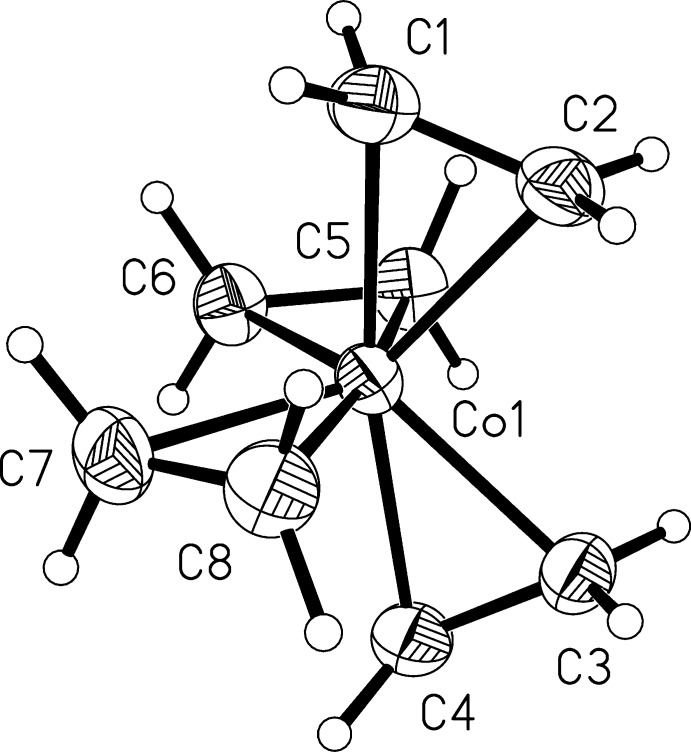
The molecular structure of the title anion, showing the atom numbering. Displacement ellipsoids are drawn at the 50% probability level.

**Fig. 2.**
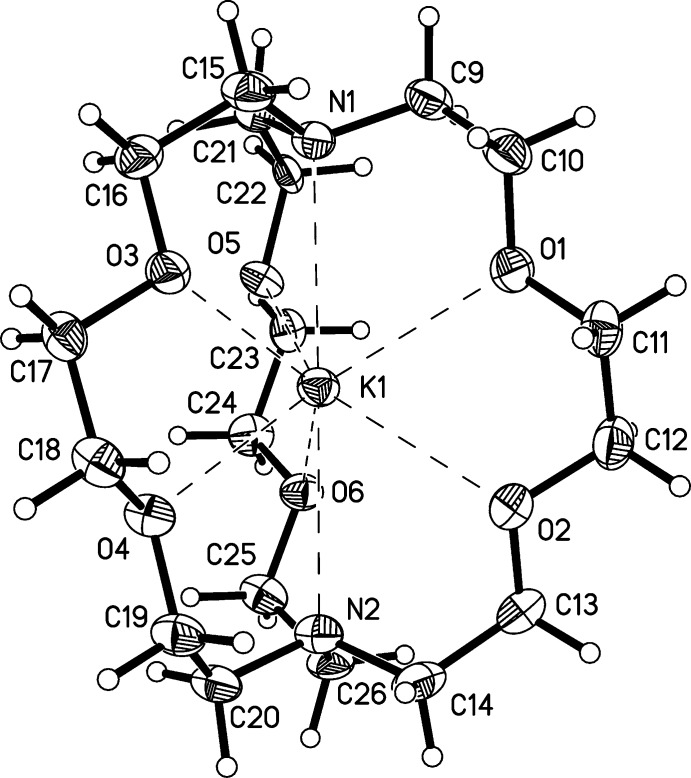
The molecular structure of the title cationic K+2.2.2-cryptand unit, showing the atom numbering. Displacement ellipsoids are drawn at the 50% probability level. Only the major fragment of the disordered arm of the 2.2.2-cryptand complexant is shown.

## supplementary crystallographic information

### Comment

The reductive synthesis of the anion from cobaltocene (CoCp_2_) with alkali metals has been reported previously in a patent (Jonas, 1979) and in review articles (Jonas & Krüger, 1980; Jonas, Schieferstein *et al.*, 1979; Jonas, 1981, 1984, 1985). Herein we report on the first structure of the title anion and the reductive synthesis from cobalt(II) bromide using potassium naphthalene as the reducing agent. Because the advantages of having cyclopentadienide (Cp^-^) as a support ligand in the reduction from CoCp_2_ (Jonas & Krüger, 1980; see discussion on page 533) were not available in our synthesis, we had to be certain that ethylene gas was present in excess, to assist naphthalene in supporting the metal center in its various oxidation states from +2 to -1. This was achieved with low temperatures, specifically 195 K, at which point ethylene appeared to be "infinitely" soluble in THF. Even at the very cold, but slightly warmer, temperature of 213 K, ethylene appeared to have finite solubility. The other interesting point in the synthesis was that naphthalene would (re)coordinate to cobalt when THF was removed under reduced pressure. Therefore the isolation of the final product required that additional ethylene gas be reintroduced to the diethyl ether slurry to displace any (re)coordinated naphthalene. It was easy to determine when the naphthalene was fully displaced because the slurry lost all trace of red and became pale yellow to off-white. Details on the isolated red naphthalenecobaltates(–I) can be found elsewhere (Brennessel *et al.*, 2006, Brennessel, 2009).

A search of the Cambridge Structural Database (CSD, Version 5.33, update No. 4, August 2012; Allen, 2002), indicated the presence of 629 structures containing an ethylene ligand, but only 29 are with first row transition metals containing at least two ethylene ligands.

The molecular structure of the title anion is illustrated in Fig. 1, and the title K^+^2.2.2-cryptand cationic unit in Fig. 2. The recently reported anion [Co(η^2^-C_2_H_4_)_2_(η^4^-C_10_H_8_)]^-^, bis(ethylene)naphthalenecobaltate(–I), which occurs twice independently as part of a triple salt (Brennessel *et al.*, 2006), has bond lengths that are statistically identical (1.410 (8) Å, avg) to those of the title complex (1.401 (6) Å, avg). The isoelectronic iron structure, [Li(tmeda)]_2_[Fe(η^2^-C_2_H_4_)_4_], has been determined with two unique C–C distances of 1.410 and 1.433 Å (Jonas, Schieferstein *et al.*, 1979); relative to that of free ethylene = 1.333 Å (Lide, 2003). No standard uncertainties were reported, but they are likely to be somewhat large (*R*1 = 7.8%). Also, the ethylene ligands have an asymmetry due to different contact distances with the lithium cations. If we take the average C–C bond length of the iron structure at face value, 1.42 Å, then it appears to be slightly longer that of the title complex, 1.401 (6) Å, which would be consistent with a more reduced metal center. All other structurally characterized homoleptic ethylene transition metal compounds are either neutral: Pt (Howard *et al.*, 1983); or cationic: Cu^+^ (Santiso-Quiñones *et al.*, 2007), Ag^+^ (Reisinger *et al.*, 2009), and Au^+^ (Dias *et al.*, 2008).

In the crystal, the cationic K^+^2.2.2-cryptand units are linked via a pair of C-H···O hydrogen bonds to form inversion dimers (Table 1). There are no other significant intermolecular interactions in the crystal structure.

### Experimental

Details on the preparation and purification of reagents and solvents, and descriptions of the equipment and techniques can be found elsewhere (Brennessel, 2009). Note that the following synthetic procedure results in a salt for which the potassium cation is complexed by 18-crown-6. Unfortunately single crystals that were grown of this complex resulted in very poor quality data (see below), and thus a different potassium complexant was incorporated for this study. To obtain the title complex the 2.2.2-cryptand salt, an aliquot of the yellow filtrate *prior to the addition of 18-crown-6*, was transferred to a flask containing excess 2.2.2-cryptand. Light yellow needles of the title complex were then grown from a pentane-layered THF solution at 273 K.

Argon was removed *in vacuo* from a flask containing deep green potassium naphthalene, K[C_10_H_8_], (13.7 mmol) in THF (50 ml, 195 K) and from a second flask containing bright blue anhydrous CoBr_2_ (1.000 g, 4.57 mmol) also in THF (50 ml, 195 K), and replaced with ethylene. At this low temperature ethylene is extremely ("infinitely") soluble and the flask system would develop a slight vacuum whenever the valve to the ethylene tank was closed. After ca. 15 psi of gas were drawn from the tank, both the tank and the flasks were closed off and argon was reintroduced to the line. Using argon pressure (a Hg bubbler was attached to the flask system to keep the pressure near 1 atm), the CoBr_2_ solution was transferred to the reducing agent *via* cannula, producing a pale yellow solution, which was then warmed slowly to room temperature (with the system open to the Hg bubbler!). The solution was filtered to remove KBr. 18-crown-6 (1.208 g, 4.57 mmol) in THF (20 ml) was added to the yellow filtrate. The solvent was removed *in vacuo*, which caused the solution to turn reddish as some naphthalene (re)coordinated to some of the product (see below). Et_2_O (75 ml) was added and argon was once again replaced with ethylene, at which point the slurry lost its red color and became nearly colorless. The lines to the flasks were freed of ethylene and replaced with argon (the flask was *not* evacuated to avoid possible re-coordination of naphthalene). The slurry was filtered, and the product was washed with Et_2_O (20 ml) and dried *in vacuo*, yielding an off-white solid (1.796 g, 83%). Although the product contained paramagnetic impurities which caused severe broadening of NMR spectral peaks, the material was sufficiently pure by this synthetic method for use in subsequent reactions. Pale yellow blocks of the18-crown-6 salt, which were grown from a pentane-layered THF solution at 273 K, were not suitable for a single-crystal X-ray experiment. The anion was badly disordered over a crystallographic twofold axis and no satisfactory model was obtained, thus the reason for the aliquot that was extracted for use with 2.2.2-cryptand to produce crystals of the title salt (see above). Crystal data for the 18-crown-6 salt: Monoclinic, *C*2/*c*; Cell constants (Å, °): *a* = 15.498 (5), *b* = 14.768 (5), *c* = 10.744 (4), *B* = 94.253 (4); *V* = 2452.2 (14) Å^3^; *Z* = 4; *T* = 173 (2) K; 2161 reflections (1813 for [*I* > 2*σ*(*I*)]).

### Refinement

One arm of the 2.2.2-cryptand complexant (atoms O5,O6,C21-C26 & O5',O6',C21'-C26') was modeled as disordered over two positions with a refined occupancy ratio of 0.559 (2):0.441 (2). Corresponding bond lengths and angles in the two orientations of the cryptand arm disorder were restrained to be similar. Anisotropic displacement parameters for spatially close atoms from the two orientations were constrained to be equivalent. H atoms on the ethylene ligands were located in a difference Fourier map and were freely refined. All other H atoms were placed geometrically and treated as riding atoms: C-H = 0.99 Å with *U*_iso(_H) = 1.2*U*_eq_(C).

### Crystal data

**Table:**

[K(C_18_H_36_N_2_O_6_)][Co(C_2_H_4_)_4_]	*F*(000) = 2528
*M**_r_* = 586.73	*D*_x_ = 1.277 Mg m^−^^3^
Orthorhombic, *P**b**c**n*	Mo *K*α radiation, λ = 0.71073 Å
Hall symbol: -P 2n 2ab	Cell parameters from 3744 reflections
*a* = 25.836 (3) Å	θ = 2.3–27.4°
*b* = 10.4820 (12) Å	µ = 0.74 mm^−^^1^
*c* = 22.544 (3) Å	*T* = 173 K
*V* = 6105.4 (12) Å^3^	Needle, light yellow
*Z* = 8	0.50 × 0.24 × 0.16 mm

### Data collection

**Table:**

Siemens SMART CCD Platform diffractometer	7010 independent reflections
Radiation source: normal-focus sealed tube	4825 reflections with *I* > 2σ(*I*)
Graphite monochromator	*R*_int_ = 0.056
ω scans	θ_max_ = 27.5°, θ_min_ = 1.6°
Absorption correction: multi-scan (*SADABS*; Sheldrick, 1996)	*h* = −26→33
*T*_min_ = 0.709, *T*_max_ = 0.891	*k* = −13→13
45000 measured reflections	*l* = −29→29

### Refinement

**Table:**

Refinement on *F*^2^	Primary atom site location: structure-invariant direct methods
Least-squares matrix: full	Secondary atom site location: difference Fourier map
*R*[*F*^2^ > 2σ(*F*^2^)] = 0.035	Hydrogen site location: inferred from neighbouring sites
*wR*(*F*^2^) = 0.073	H atoms treated by a mixture of independent and constrained refinement
*S* = 1.02	*w* = 1/[σ^2^(*F*_o_^2^) + (0.0242*P*)^2^ + 2.9795*P*] where *P* = (*F*_o_^2^ + 2*F*_c_^2^)/3
7010 reflections	(Δ/σ)_max_ = 0.001
414 parameters	Δρ_max_ = 0.31 e Å^−^^3^
16 restraints	Δρ_min_ = −0.31 e Å^−^^3^

### Special details

**Table:**

Geometry. All e.s.d.'s (except the e.s.d. in the dihedral angle between two l.s. planes) are estimated using the full covariance matrix. The cell e.s.d.'s are taken into account individually in the estimation of e.s.d.'s in distances, angles and torsion angles; correlations between e.s.d.'s in cell parameters are only used when they are defined by crystal symmetry. An approximate (isotropic) treatment of cell e.s.d.'s is used for estimating e.s.d.'s involving l.s. planes.
Refinement. Refinement of *F*^2^ against ALL reflections. The weighted *R*-factor *wR* and goodness of fit *S* are based on *F*^2^, conventional *R*-factors *R* are based on *F*, with *F* set to zero for negative *F*^2^. The threshold expression of *F*^2^ > σ(*F*^2^) is used only for calculating *R*-factors(gt) *etc*. and is not relevant to the choice of reflections for refinement. *R*-factors based on *F*^2^ are statistically about twice as large as those based on *F*, and *R*- factors based on ALL data will be even larger.

### Fractional atomic coordinates and isotropic or equivalent isotropic displacement parameters (Å^2^)

**Table:**

	*x*	*y*	*z*	*U*_iso_*/*U*_eq_	Occ. (<1)
Co1	0.371067 (10)	0.52565 (3)	0.455752 (11)	0.02568 (7)	
C1	0.40825 (9)	0.6890 (2)	0.43378 (11)	0.0369 (5)	
H1A	0.3861 (8)	0.750 (2)	0.4136 (9)	0.033 (6)*	
H1B	0.4258 (8)	0.725 (2)	0.4690 (9)	0.042 (6)*	
C2	0.43103 (9)	0.5863 (2)	0.40416 (11)	0.0368 (5)	
H2A	0.4231 (8)	0.574 (2)	0.3623 (10)	0.042 (6)*	
H2B	0.4641 (8)	0.553 (2)	0.4181 (9)	0.039 (6)*	
C3	0.38604 (9)	0.3426 (2)	0.43062 (10)	0.0350 (5)	
H3A	0.3803 (8)	0.330 (2)	0.3862 (10)	0.044 (6)*	
H3B	0.4191 (8)	0.316 (2)	0.4458 (9)	0.035 (6)*	
H4B	0.3467 (9)	0.321 (2)	0.5106 (10)	0.050 (7)*	
C4	0.34319 (9)	0.3457 (2)	0.46850 (11)	0.0376 (5)	
H4A	0.3074 (9)	0.333 (2)	0.4523 (10)	0.051 (7)*	
C5	0.31747 (8)	0.5552 (2)	0.39157 (10)	0.0351 (5)	
H5A	0.3026 (8)	0.476 (2)	0.3784 (9)	0.037 (6)*	
H5B	0.3302 (9)	0.615 (2)	0.3607 (10)	0.051 (7)*	
C6	0.29971 (8)	0.6068 (2)	0.44527 (9)	0.0347 (5)	
H6A	0.2733 (9)	0.562 (2)	0.4666 (10)	0.048 (7)*	
H6B	0.2998 (8)	0.701 (2)	0.4511 (9)	0.043 (6)*	
C7	0.36906 (10)	0.5658 (3)	0.54443 (10)	0.0417 (5)	
H7A	0.3434 (9)	0.513 (2)	0.5645 (10)	0.047 (7)*	
H7B	0.3685 (9)	0.656 (3)	0.5526 (10)	0.057 (7)*	
C8	0.41614 (10)	0.5080 (3)	0.52897 (9)	0.0402 (5)	
H8A	0.4221 (9)	0.417 (2)	0.5389 (10)	0.051 (7)*	
H8B	0.4488 (9)	0.559 (2)	0.5242 (10)	0.050 (7)*	
K1	0.382354 (15)	0.89809 (4)	0.672690 (17)	0.02532 (10)	
N1	0.30312 (6)	0.72674 (15)	0.72648 (7)	0.0258 (4)	
C9	0.26926 (8)	0.67901 (19)	0.67875 (9)	0.0322 (5)	
H9A	0.2389	0.6366	0.6969	0.039*	
H9B	0.2884	0.6140	0.6556	0.039*	
C10	0.25047 (8)	0.7816 (2)	0.63696 (9)	0.0349 (5)	
H10A	0.2265	0.7445	0.6074	0.042*	
H10B	0.2317	0.8483	0.6594	0.042*	
O1	0.29389 (5)	0.83542 (13)	0.60803 (6)	0.0318 (3)	
C11	0.27992 (8)	0.9181 (2)	0.56039 (9)	0.0347 (5)	
H11A	0.2643	0.9973	0.5762	0.042*	
H11B	0.2541	0.8758	0.5345	0.042*	
C12	0.32733 (8)	0.9491 (2)	0.52562 (9)	0.0352 (5)	
H12A	0.3436	0.8695	0.5111	0.042*	
H12B	0.3180	1.0019	0.4908	0.042*	
O2	0.36279 (5)	1.01677 (13)	0.56228 (6)	0.0322 (3)	
C13	0.40865 (8)	1.0484 (2)	0.53034 (9)	0.0377 (5)	
H13A	0.3995	1.0927	0.4930	0.045*	
H13B	0.4278	0.9697	0.5202	0.045*	
C14	0.44192 (9)	1.1334 (2)	0.56788 (9)	0.0381 (5)	
H14A	0.4707	1.1663	0.5433	0.046*	
H14B	0.4211	1.2074	0.5810	0.046*	
C15	0.27290 (8)	0.7955 (2)	0.77164 (9)	0.0329 (5)	
H15A	0.2549	0.7324	0.7969	0.039*	
H15B	0.2461	0.8473	0.7514	0.039*	
C16	0.30469 (8)	0.8818 (2)	0.81075 (8)	0.0322 (5)	
H16A	0.2826	0.9185	0.8423	0.039*	
H16B	0.3328	0.8324	0.8298	0.039*	
O3	0.32619 (5)	0.98158 (12)	0.77543 (5)	0.0280 (3)	
C17	0.35132 (7)	1.07411 (19)	0.81130 (8)	0.0289 (4)	
H17A	0.3793	1.0331	0.8345	0.035*	
H17B	0.3262	1.1117	0.8395	0.035*	
C18	0.37352 (7)	1.17667 (18)	0.77276 (9)	0.0305 (4)	
H18A	0.3457	1.2154	0.7485	0.037*	
H18B	0.3889	1.2444	0.7978	0.037*	
O4	0.41232 (5)	1.12393 (12)	0.73491 (6)	0.0272 (3)	
C19	0.43554 (8)	1.22063 (19)	0.69949 (9)	0.0319 (5)	
H19A	0.4483	1.2903	0.7253	0.038*	
H19B	0.4095	1.2567	0.6720	0.038*	
C20	0.47980 (8)	1.16543 (19)	0.66462 (9)	0.0308 (5)	
H20A	0.4981	1.2355	0.6440	0.037*	
H20B	0.5046	1.1255	0.6925	0.037*	
C21	0.3328 (5)	0.6257 (11)	0.7567 (7)	0.0261 (18)	0.559 (2)
H21A	0.3082	0.5586	0.7692	0.031*	0.559 (2)
H21B	0.3481	0.6627	0.7931	0.031*	0.559 (2)
C22	0.3756 (5)	0.5629 (14)	0.7221 (6)	0.0234 (17)	0.559 (2)
H22A	0.3893	0.4889	0.7444	0.028*	0.559 (2)
H22B	0.3621	0.5318	0.6836	0.028*	0.559 (2)
O5	0.4154 (2)	0.6533 (7)	0.7125 (2)	0.0263 (9)	0.559 (2)
C23	0.4579 (3)	0.5979 (8)	0.6814 (4)	0.0259 (15)	0.559 (2)
H23A	0.4469	0.5723	0.6411	0.031*	0.559 (2)
H23B	0.4702	0.5210	0.7026	0.031*	0.559 (2)
C24	0.50011 (14)	0.6945 (3)	0.67784 (17)	0.0326 (7)	0.559 (2)
H24A	0.5098	0.7223	0.7183	0.039*	0.559 (2)
H24B	0.5310	0.6559	0.6590	0.039*	0.559 (2)
O6	0.48329 (11)	0.8030 (3)	0.64367 (13)	0.0277 (5)	0.559 (2)
C25	0.5242 (4)	0.8941 (10)	0.6412 (3)	0.0303 (16)	0.559 (2)
H25A	0.5566	0.8526	0.6279	0.036*	0.559 (2)
H25B	0.5301	0.9313	0.6810	0.036*	0.559 (2)
C26	0.5087 (5)	0.9973 (14)	0.5980 (6)	0.0299 (16)	0.559 (2)
H26A	0.5382	1.0562	0.5919	0.036*	0.559 (2)
H26B	0.5000	0.9584	0.5593	0.036*	0.559 (2)
C21'	0.3271 (7)	0.6114 (14)	0.7518 (9)	0.0261 (18)	0.441 (2)
H21C	0.3000	0.5464	0.7586	0.031*	0.441 (2)
H21D	0.3426	0.6329	0.7907	0.031*	0.441 (2)
C22'	0.3683 (6)	0.5561 (19)	0.7124 (8)	0.0234 (17)	0.441 (2)
H22C	0.3863	0.4863	0.7335	0.028*	0.441 (2)
H22D	0.3520	0.5199	0.6763	0.028*	0.441 (2)
O5'	0.4041 (3)	0.6504 (10)	0.6961 (3)	0.0263 (9)	0.441 (2)
C23'	0.4495 (4)	0.6008 (11)	0.6690 (6)	0.0259 (15)	0.441 (2)
H23C	0.4398	0.5327	0.6407	0.031*	0.441 (2)
H23D	0.4721	0.5626	0.6998	0.031*	0.441 (2)
C24'	0.47835 (18)	0.7025 (4)	0.6372 (2)	0.0326 (7)	0.441 (2)
H24C	0.5124	0.6698	0.6241	0.039*	0.441 (2)
H24D	0.4587	0.7297	0.6016	0.039*	0.441 (2)
O6'	0.48526 (15)	0.8071 (4)	0.67589 (16)	0.0277 (5)	0.441 (2)
C25'	0.5240 (6)	0.8960 (13)	0.6595 (5)	0.0303 (16)	0.441 (2)
H25C	0.5553	0.8487	0.6471	0.036*	0.441 (2)
H25D	0.5332	0.9478	0.6947	0.036*	0.441 (2)
C26'	0.5084 (7)	0.9842 (18)	0.6102 (8)	0.0299 (16)	0.441 (2)
H26C	0.5386	1.0381	0.6003	0.036*	0.441 (2)
H26D	0.5006	0.9315	0.5749	0.036*	0.441 (2)
N2	0.46366 (6)	1.06963 (15)	0.62048 (7)	0.0283 (4)	

### Atomic displacement parameters (Å^2^)

**Table:**

	*U*^11^	*U*^22^	*U*^33^	*U*^12^	*U*^13^	*U*^23^
Co1	0.02499 (13)	0.02911 (14)	0.02294 (12)	0.00124 (12)	0.00108 (11)	0.00137 (11)
C1	0.0348 (12)	0.0341 (13)	0.0419 (12)	−0.0013 (11)	0.0019 (10)	0.0056 (11)
C2	0.0314 (12)	0.0367 (13)	0.0424 (13)	−0.0013 (10)	0.0076 (10)	0.0070 (11)
C3	0.0348 (12)	0.0317 (12)	0.0386 (12)	0.0033 (10)	0.0024 (10)	−0.0023 (10)
C4	0.0380 (13)	0.0319 (12)	0.0427 (13)	−0.0046 (10)	0.0082 (11)	0.0028 (10)
C5	0.0298 (11)	0.0423 (14)	0.0331 (12)	0.0044 (10)	−0.0051 (9)	−0.0006 (11)
C6	0.0283 (11)	0.0412 (14)	0.0344 (12)	0.0045 (10)	−0.0009 (9)	−0.0004 (10)
C7	0.0523 (14)	0.0483 (15)	0.0246 (10)	0.0059 (13)	−0.0019 (11)	−0.0014 (11)
C8	0.0454 (14)	0.0433 (15)	0.0319 (11)	0.0078 (12)	−0.0087 (10)	0.0016 (10)
K1	0.0239 (2)	0.0239 (2)	0.0281 (2)	−0.00110 (17)	−0.00141 (16)	0.00295 (17)
N1	0.0244 (8)	0.0249 (9)	0.0281 (8)	−0.0032 (7)	0.0024 (7)	0.0000 (7)
C9	0.0293 (11)	0.0307 (11)	0.0367 (11)	−0.0084 (9)	−0.0001 (9)	−0.0017 (9)
C10	0.0254 (10)	0.0391 (13)	0.0402 (11)	−0.0048 (9)	−0.0034 (10)	−0.0004 (10)
O1	0.0244 (7)	0.0400 (8)	0.0309 (7)	−0.0002 (6)	−0.0043 (6)	0.0062 (6)
C11	0.0318 (11)	0.0420 (13)	0.0303 (10)	0.0057 (10)	−0.0093 (9)	0.0019 (9)
C12	0.0359 (12)	0.0434 (13)	0.0262 (10)	0.0025 (10)	−0.0062 (9)	0.0028 (9)
O2	0.0347 (8)	0.0374 (8)	0.0245 (6)	−0.0034 (7)	0.0014 (6)	0.0010 (6)
C13	0.0437 (13)	0.0424 (14)	0.0270 (11)	−0.0052 (11)	0.0047 (9)	0.0082 (9)
C14	0.0444 (13)	0.0348 (13)	0.0352 (11)	−0.0078 (10)	0.0070 (10)	0.0105 (10)
C15	0.0259 (10)	0.0334 (12)	0.0394 (12)	−0.0047 (9)	0.0096 (9)	−0.0046 (10)
C16	0.0326 (11)	0.0352 (12)	0.0289 (10)	−0.0037 (9)	0.0082 (8)	−0.0031 (9)
O3	0.0297 (7)	0.0270 (7)	0.0272 (7)	−0.0049 (6)	0.0025 (5)	−0.0044 (6)
C17	0.0247 (10)	0.0312 (11)	0.0307 (10)	0.0008 (9)	0.0022 (8)	−0.0103 (9)
C18	0.0255 (10)	0.0263 (10)	0.0398 (11)	0.0014 (9)	0.0052 (9)	−0.0118 (9)
O4	0.0243 (7)	0.0221 (7)	0.0353 (7)	0.0012 (6)	0.0064 (6)	−0.0043 (6)
C19	0.0334 (11)	0.0215 (10)	0.0409 (12)	−0.0031 (9)	0.0044 (9)	−0.0011 (9)
C20	0.0298 (11)	0.0245 (11)	0.0382 (11)	−0.0070 (9)	0.0062 (9)	−0.0023 (9)
C21	0.030 (3)	0.021 (2)	0.027 (2)	−0.012 (2)	−0.0007 (19)	0.002 (2)
C22	0.028 (3)	0.0190 (16)	0.023 (4)	−0.0051 (18)	−0.010 (3)	−0.001 (2)
O5	0.022 (2)	0.0204 (8)	0.037 (3)	−0.0026 (17)	−0.0008 (16)	−0.002 (2)
C23	0.025 (3)	0.0245 (11)	0.028 (4)	0.0080 (16)	−0.007 (2)	0.0018 (18)
C24	0.0266 (16)	0.0325 (16)	0.0387 (18)	0.0046 (13)	0.0004 (12)	0.0030 (15)
O6	0.0253 (9)	0.0248 (9)	0.0328 (15)	0.0002 (7)	0.0010 (15)	0.0038 (15)
C25	0.0225 (11)	0.0288 (12)	0.040 (5)	−0.0003 (10)	0.009 (3)	−0.010 (3)
C26	0.0272 (11)	0.032 (3)	0.030 (5)	−0.0075 (15)	0.014 (2)	−0.002 (3)
C21'	0.030 (3)	0.021 (2)	0.027 (2)	−0.012 (2)	−0.0007 (19)	0.002 (2)
C22'	0.028 (3)	0.0190 (16)	0.023 (4)	−0.0051 (18)	−0.010 (3)	−0.001 (2)
O5'	0.022 (2)	0.0204 (8)	0.037 (3)	−0.0026 (17)	−0.0008 (16)	−0.002 (2)
C23'	0.025 (3)	0.0245 (11)	0.028 (4)	0.0080 (16)	−0.007 (2)	0.0018 (18)
C24'	0.0266 (16)	0.0325 (16)	0.0387 (18)	0.0046 (13)	0.0004 (12)	0.0030 (15)
O6'	0.0253 (9)	0.0248 (9)	0.0328 (15)	0.0002 (7)	0.0010 (15)	0.0038 (15)
C25'	0.0225 (11)	0.0288 (12)	0.040 (5)	−0.0003 (10)	0.009 (3)	−0.010 (3)
C26'	0.0272 (11)	0.032 (3)	0.030 (5)	−0.0075 (15)	0.014 (2)	−0.002 (3)
N2	0.0277 (9)	0.0243 (9)	0.0329 (9)	−0.0019 (7)	0.0063 (7)	−0.0009 (7)

### Geometric parameters (Å, º)

**Table:**

Co1—C1	2.025 (2)	C14—H14B	0.9900
Co1—C5	2.027 (2)	C15—C16	1.506 (3)
Co1—C8	2.029 (2)	C15—H15A	0.9900
Co1—C3	2.038 (2)	C15—H15B	0.9900
Co1—C2	2.039 (2)	C16—O3	1.427 (2)
Co1—C4	2.039 (2)	C16—H16A	0.9900
Co1—C7	2.044 (2)	C16—H16B	0.9900
Co1—C6	2.044 (2)	O3—C17	1.420 (2)
C1—C2	1.397 (3)	C17—C18	1.497 (3)
C1—H1A	0.97 (2)	C17—H17A	0.9900
C1—H1B	0.99 (2)	C17—H17B	0.9900
C2—H2A	0.98 (2)	C18—O4	1.428 (2)
C2—H2B	0.97 (2)	C18—H18A	0.9900
C3—C4	1.399 (3)	C18—H18B	0.9900
C3—H3A	1.02 (2)	O4—C19	1.423 (2)
C3—H3B	0.96 (2)	C19—C20	1.503 (3)
C4—H4B	0.99 (2)	C19—H19A	0.9900
C4—H4A	1.00 (2)	C19—H19B	0.9900
C5—C6	1.403 (3)	C20—N2	1.474 (2)
C5—H5A	0.96 (2)	C20—H20A	0.9900
C5—H5B	1.00 (2)	C20—H20B	0.9900
C6—H6A	0.96 (2)	C21—C22	1.504 (9)
C6—H6B	0.99 (2)	C21—H21A	0.9900
C7—C8	1.403 (3)	C21—H21B	0.9900
C7—H7A	0.97 (2)	C22—O5	1.417 (8)
C7—H7B	0.96 (3)	C22—H22A	0.9900
C8—H8A	0.99 (2)	C22—H22B	0.9900
C8—H8B	1.01 (2)	O5—C23	1.425 (8)
K1—O5'	2.709 (10)	C23—C24	1.491 (8)
K1—O1	2.7893 (13)	C23—H23A	0.9900
K1—O6'	2.825 (4)	C23—H23B	0.9900
K1—O2	2.8282 (14)	C24—O6	1.441 (4)
K1—O5	2.849 (7)	C24—H24A	0.9900
K1—O4	2.8586 (13)	C24—H24B	0.9900
K1—O6	2.867 (3)	O6—C25	1.425 (8)
K1—O3	2.8700 (13)	C25—C26	1.509 (8)
K1—N1	2.9811 (16)	C25—H25A	0.9900
K1—N2	3.0052 (16)	C25—H25B	0.9900
K1—C24'	3.316 (5)	C26—N2	1.479 (3)
N1—C15	1.472 (2)	C26—H26A	0.9900
N1—C21'	1.474 (3)	C26—H26B	0.9900
N1—C21	1.474 (3)	C21'—C22'	1.502 (11)
N1—C9	1.474 (2)	C21'—H21C	0.9900
C9—C10	1.510 (3)	C21'—H21D	0.9900
C9—H9A	0.9900	C22'—O5'	1.404 (11)
C9—H9B	0.9900	C22'—H22C	0.9900
C10—O1	1.415 (2)	C22'—H22D	0.9900
C10—H10A	0.9900	O5'—C23'	1.420 (10)
C10—H10B	0.9900	C23'—C24'	1.486 (10)
O1—C11	1.427 (2)	C23'—H23C	0.9900
C11—C12	1.490 (3)	C23'—H23D	0.9900
C11—H11A	0.9900	C24'—O6'	1.413 (6)
C11—H11B	0.9900	C24'—H24C	0.9900
C12—O2	1.423 (2)	C24'—H24D	0.9900
C12—H12A	0.9900	O6'—C25'	1.416 (10)
C12—H12B	0.9900	C25'—C26'	1.500 (11)
O2—C13	1.426 (2)	C25'—H25C	0.9900
C13—C14	1.499 (3)	C25'—H25D	0.9900
C13—H13A	0.9900	C26'—N2	1.479 (3)
C13—H13B	0.9900	C26'—H26C	0.9900
C14—N2	1.473 (3)	C26'—H26D	0.9900
C14—H14A	0.9900		
			
C1—Co1—C5	91.15 (10)	O2—C12—C11	109.41 (16)
C1—Co1—C8	90.22 (11)	O2—C12—H12A	109.8
C5—Co1—C8	170.81 (9)	C11—C12—H12A	109.8
C1—Co1—C3	129.66 (9)	O2—C12—H12B	109.8
C5—Co1—C3	94.31 (10)	C11—C12—H12B	109.8
C8—Co1—C3	91.80 (10)	H12A—C12—H12B	108.2
C1—Co1—C2	40.20 (9)	C12—O2—C13	110.95 (15)
C5—Co1—C2	93.68 (10)	C12—O2—K1	114.01 (11)
C8—Co1—C2	93.23 (10)	C13—O2—K1	113.47 (11)
C3—Co1—C2	89.48 (9)	O2—C13—C14	109.25 (16)
C1—Co1—C4	169.78 (9)	O2—C13—H13A	109.8
C5—Co1—C4	90.04 (10)	C14—C13—H13A	109.8
C8—Co1—C4	90.22 (11)	O2—C13—H13B	109.8
C3—Co1—C4	40.13 (9)	C14—C13—H13B	109.8
C2—Co1—C4	129.59 (9)	H13A—C13—H13B	108.3
C1—Co1—C7	94.43 (11)	N2—C14—C13	113.81 (17)
C5—Co1—C7	130.51 (9)	N2—C14—H14A	108.8
C8—Co1—C7	40.31 (9)	C13—C14—H14A	108.8
C3—Co1—C7	118.08 (10)	N2—C14—H14B	108.8
C2—Co1—C7	120.87 (11)	C13—C14—H14B	108.8
C4—Co1—C7	92.50 (10)	H14A—C14—H14B	107.7
C1—Co1—C6	92.73 (10)	N1—C15—C16	114.18 (16)
C5—Co1—C6	40.32 (8)	N1—C15—H15A	108.7
C8—Co1—C6	130.53 (9)	C16—C15—H15A	108.7
C3—Co1—C6	122.07 (10)	N1—C15—H15B	108.7
C2—Co1—C6	119.31 (10)	C16—C15—H15B	108.7
C4—Co1—C6	94.74 (10)	H15A—C15—H15B	107.6
C7—Co1—C6	90.25 (9)	O3—C16—C15	109.01 (15)
C2—C1—Co1	70.43 (13)	O3—C16—H16A	109.9
C2—C1—H1A	122.1 (12)	C15—C16—H16A	109.9
Co1—C1—H1A	113.0 (12)	O3—C16—H16B	109.9
C2—C1—H1B	119.0 (13)	C15—C16—H16B	109.9
Co1—C1—H1B	110.1 (12)	H16A—C16—H16B	108.3
H1A—C1—H1B	113.4 (17)	C17—O3—C16	111.16 (14)
C1—C2—Co1	69.37 (12)	C17—O3—K1	115.90 (10)
C1—C2—H2A	118.6 (13)	C16—O3—K1	115.05 (10)
Co1—C2—H2A	110.5 (13)	O3—C17—C18	109.59 (15)
C1—C2—H2B	119.3 (12)	O3—C17—H17A	109.8
Co1—C2—H2B	111.9 (12)	C18—C17—H17A	109.8
H2A—C2—H2B	116.7 (18)	O3—C17—H17B	109.8
C4—C3—Co1	69.98 (13)	C18—C17—H17B	109.8
C4—C3—H3A	119.2 (12)	H17A—C17—H17B	108.2
Co1—C3—H3A	111.3 (12)	O4—C18—C17	109.76 (15)
C4—C3—H3B	119.4 (12)	O4—C18—H18A	109.7
Co1—C3—H3B	110.2 (12)	C17—C18—H18A	109.7
H3A—C3—H3B	116.2 (17)	O4—C18—H18B	109.7
C3—C4—Co1	69.89 (13)	C17—C18—H18B	109.7
C3—C4—H4B	120.5 (13)	H18A—C18—H18B	108.2
Co1—C4—H4B	110.2 (14)	C19—O4—C18	110.83 (14)
C3—C4—H4A	120.3 (13)	C19—O4—K1	115.38 (11)
Co1—C4—H4A	113.3 (13)	C18—O4—K1	115.08 (10)
H4B—C4—H4A	113.6 (18)	O4—C19—C20	109.88 (16)
C6—C5—Co1	70.50 (12)	O4—C19—H19A	109.7
C6—C5—H5A	117.9 (12)	C20—C19—H19A	109.7
Co1—C5—H5A	111.3 (12)	O4—C19—H19B	109.7
C6—C5—H5B	117.8 (13)	C20—C19—H19B	109.7
Co1—C5—H5B	111.7 (13)	H19A—C19—H19B	108.2
H5A—C5—H5B	117.6 (18)	N2—C20—C19	113.58 (16)
C5—C6—Co1	69.17 (12)	N2—C20—H20A	108.8
C5—C6—H6A	118.7 (13)	C19—C20—H20A	108.8
Co1—C6—H6A	112.5 (13)	N2—C20—H20B	108.8
C5—C6—H6B	119.7 (12)	C19—C20—H20B	108.8
Co1—C6—H6B	113.3 (12)	H20A—C20—H20B	107.7
H6A—C6—H6B	114.7 (18)	N1—C21—C22	117.2 (11)
C8—C7—Co1	69.27 (13)	N1—C21—H21A	108.0
C8—C7—H7A	117.6 (13)	C22—C21—H21A	108.0
Co1—C7—H7A	110.8 (13)	N1—C21—H21B	108.0
C8—C7—H7B	118.9 (15)	C22—C21—H21B	108.0
Co1—C7—H7B	113.0 (14)	H21A—C21—H21B	107.2
H7A—C7—H7B	117 (2)	O5—C22—C21	108.7 (11)
C7—C8—Co1	70.42 (13)	O5—C22—H22A	110.0
C7—C8—H8A	119.6 (13)	C21—C22—H22A	110.0
Co1—C8—H8A	111.1 (13)	O5—C22—H22B	110.0
C7—C8—H8B	121.6 (13)	C21—C22—H22B	110.0
Co1—C8—H8B	110.3 (13)	H22A—C22—H22B	108.3
H8A—C8—H8B	114.0 (19)	C22—O5—C23	111.2 (8)
O5'—K1—O1	92.66 (13)	C22—O5—K1	115.6 (7)
O5'—K1—O6'	58.41 (18)	C23—O5—K1	116.3 (5)
O1—K1—O6'	134.83 (8)	O5—C23—C24	108.2 (7)
O5'—K1—O2	129.08 (15)	O5—C23—H23A	110.1
O1—K1—O2	59.82 (4)	C24—C23—H23A	110.1
O6'—K1—O2	109.82 (8)	O5—C23—H23B	110.1
O5'—K1—O5	9.30 (18)	C24—C23—H23B	110.1
O1—K1—O5	101.45 (10)	H23A—C23—H23B	108.4
O6'—K1—O5	53.54 (14)	O6—C24—C23	110.1 (4)
O2—K1—O5	136.63 (12)	O6—C24—H24A	109.6
O5'—K1—O4	129.93 (12)	C23—C24—H24A	109.6
O1—K1—O4	132.33 (4)	O6—C24—H24B	109.6
O6'—K1—O4	90.69 (8)	C23—C24—H24B	109.6
O2—K1—O4	96.66 (4)	H24A—C24—H24B	108.1
O5—K1—O4	120.65 (10)	C25—O6—C24	109.1 (5)
O5'—K1—O6	61.47 (19)	C25—O6—K1	116.7 (5)
O1—K1—O6	122.97 (7)	C24—O6—K1	115.2 (2)
O6'—K1—O6	14.70 (6)	O6—C25—C26	108.1 (10)
O2—K1—O6	96.58 (6)	O6—C25—H25A	110.1
O5—K1—O6	59.07 (14)	C26—C25—H25A	110.1
O4—K1—O6	98.83 (6)	O6—C25—H25B	110.1
O5'—K1—O3	103.89 (19)	C26—C25—H25B	110.1
O1—K1—O3	94.55 (4)	H25A—C25—H25B	108.4
O6'—K1—O3	123.92 (8)	N2—C26—C25	110.7 (8)
O2—K1—O3	119.06 (4)	N2—C26—H26A	109.5
O5—K1—O3	99.91 (13)	C25—C26—H26A	109.5
O4—K1—O3	59.22 (4)	N2—C26—H26B	109.5
O6—K1—O3	138.59 (6)	C25—C26—H26B	109.5
O5'—K1—N1	59.06 (18)	H26A—C26—H26B	108.1
O1—K1—N1	60.53 (4)	N1—C21'—C22'	112.7 (15)
O6'—K1—N1	115.63 (8)	N1—C21'—H21C	109.1
O2—K1—N1	120.02 (4)	C22'—C21'—H21C	109.1
O5—K1—N1	62.32 (13)	N1—C21'—H21D	109.1
O4—K1—N1	119.03 (4)	C22'—C21'—H21D	109.1
O6—K1—N1	120.53 (7)	H21C—C21'—H21D	107.8
O3—K1—N1	60.55 (4)	O5'—C22'—C21'	110.5 (14)
O5'—K1—N2	120.31 (18)	O5'—C22'—H22C	109.6
O1—K1—N2	120.60 (4)	C21'—C22'—H22C	109.6
O6'—K1—N2	63.52 (8)	O5'—C22'—H22D	109.6
O2—K1—N2	61.12 (4)	C21'—C22'—H22D	109.6
O5—K1—N2	116.90 (13)	H22C—C22'—H22D	108.1
O4—K1—N2	60.49 (4)	C22'—O5'—C23'	113.6 (11)
O6—K1—N2	58.86 (7)	C22'—O5'—K1	126.1 (10)
O3—K1—N2	119.15 (4)	C23'—O5'—K1	116.0 (6)
N1—K1—N2	178.86 (5)	O5'—C23'—C24'	111.0 (9)
O5'—K1—C24'	45.5 (2)	O5'—C23'—H23C	109.4
O1—K1—C24'	109.94 (9)	C24'—C23'—H23C	109.4
O6'—K1—C24'	25.00 (11)	O5'—C23'—H23D	109.4
O2—K1—C24'	101.13 (9)	C24'—C23'—H23D	109.4
O5—K1—C24'	45.17 (16)	H23C—C23'—H23D	108.0
O4—K1—C24'	115.34 (9)	O6'—C24'—C23'	108.8 (6)
O6—K1—C24'	18.15 (9)	O6'—C24'—K1	57.7 (2)
O3—K1—C24'	139.61 (9)	C23'—C24'—K1	87.2 (5)
N1—K1—C24'	103.85 (9)	O6'—C24'—H24C	109.9
N2—K1—C24'	75.67 (9)	C23'—C24'—H24C	109.9
C15—N1—C21'	110.9 (8)	K1—C24'—H24C	162.1
C15—N1—C21	108.0 (6)	O6'—C24'—H24D	109.9
C15—N1—C9	110.88 (15)	C23'—C24'—H24D	109.9
C21'—N1—C9	104.7 (10)	K1—C24'—H24D	68.5
C21—N1—C9	113.7 (7)	H24C—C24'—H24D	108.3
C15—N1—K1	110.52 (11)	C24'—O6'—C25'	116.0 (6)
C21'—N1—K1	111.3 (7)	C24'—O6'—K1	97.3 (3)
C21—N1—K1	105.3 (6)	C25'—O6'—K1	115.8 (7)
C9—N1—K1	108.37 (11)	O6'—C25'—C26'	114.2 (13)
N1—C9—C10	113.87 (16)	O6'—C25'—H25C	108.7
N1—C9—H9A	108.8	C26'—C25'—H25C	108.7
C10—C9—H9A	108.8	O6'—C25'—H25D	108.7
N1—C9—H9B	108.8	C26'—C25'—H25D	108.7
C10—C9—H9B	108.8	H25C—C25'—H25D	107.6
H9A—C9—H9B	107.7	N2—C26'—C25'	117.8 (11)
O1—C10—C9	108.46 (16)	N2—C26'—H26C	107.9
O1—C10—H10A	110.0	C25'—C26'—H26C	107.9
C9—C10—H10A	110.0	N2—C26'—H26D	107.9
O1—C10—H10B	110.0	C25'—C26'—H26D	107.9
C9—C10—H10B	110.0	H26C—C26'—H26D	107.2
H10A—C10—H10B	108.4	C14—N2—C20	110.02 (16)
C10—O1—C11	112.87 (15)	C14—N2—C26	104.9 (5)
C10—O1—K1	120.18 (11)	C20—N2—C26	111.0 (8)
C11—O1—K1	117.25 (11)	C14—N2—C26'	116.5 (7)
O1—C11—C12	108.69 (16)	C20—N2—C26'	107.3 (11)
O1—C11—H11A	110.0	C14—N2—K1	108.69 (11)
C12—C11—H11A	110.0	C20—N2—K1	109.94 (11)
O1—C11—H11B	110.0	C26—N2—K1	112.2 (6)
C12—C11—H11B	110.0	C26'—N2—K1	104.2 (8)
H11A—C11—H11B	108.3		
			
C5—Co1—C1—C2	−94.34 (15)	O6—K1—O4—C18	−160.64 (13)
C8—Co1—C1—C2	94.75 (16)	O3—K1—O4—C18	−18.37 (11)
C3—Co1—C1—C2	2.2 (2)	N1—K1—O4—C18	−28.25 (13)
C4—Co1—C1—C2	2.3 (6)	N2—K1—O4—C18	152.94 (13)
C7—Co1—C1—C2	134.88 (16)	C24'—K1—O4—C18	−152.70 (14)
C6—Co1—C1—C2	−134.65 (15)	C18—O4—C19—C20	174.28 (16)
C5—Co1—C2—C1	87.43 (16)	K1—O4—C19—C20	−52.68 (18)
C8—Co1—C2—C1	−86.51 (16)	O4—C19—C20—N2	65.2 (2)
C3—Co1—C2—C1	−178.28 (16)	C15—N1—C21—C22	−166.3 (9)
C4—Co1—C2—C1	−179.46 (15)	C9—N1—C21—C22	70.3 (12)
C7—Co1—C2—C1	−55.39 (18)	K1—N1—C21—C22	−48.2 (13)
C6—Co1—C2—C1	54.58 (18)	N1—C21—C22—O5	67.7 (15)
C1—Co1—C3—C4	179.97 (15)	C21—C22—O5—C23	178.2 (9)
C5—Co1—C3—C4	−84.93 (16)	C21—C22—O5—K1	−46.3 (11)
C8—Co1—C3—C4	88.20 (16)	O5'—K1—O5—C22	−50.1 (19)
C2—Co1—C3—C4	−178.58 (16)	O1—K1—O5—C22	−30.7 (7)
C7—Co1—C3—C4	56.20 (18)	O6'—K1—O5—C22	−168.8 (7)
C6—Co1—C3—C4	−53.70 (18)	O2—K1—O5—C22	−88.5 (7)
C1—Co1—C4—C3	−0.1 (6)	O4—K1—O5—C22	126.0 (6)
C5—Co1—C4—C3	96.65 (15)	O6—K1—O5—C22	−152.4 (7)
C8—Co1—C4—C3	−92.54 (15)	O3—K1—O5—C22	66.0 (6)
C2—Co1—C4—C3	1.8 (2)	N1—K1—O5—C22	17.0 (6)
C7—Co1—C4—C3	−132.79 (15)	N2—K1—O5—C22	−164.0 (6)
C6—Co1—C4—C3	136.74 (15)	C24'—K1—O5—C22	−137.5 (7)
C1—Co1—C5—C6	−92.86 (16)	O5'—K1—O5—C23	83 (2)
C8—Co1—C5—C6	5.7 (7)	O1—K1—O5—C23	102.5 (4)
C3—Co1—C5—C6	137.23 (15)	O6'—K1—O5—C23	−35.7 (4)
C2—Co1—C5—C6	−133.02 (16)	O2—K1—O5—C23	44.6 (5)
C4—Co1—C5—C6	97.29 (16)	O4—K1—O5—C23	−100.8 (4)
C7—Co1—C5—C6	4.0 (2)	O6—K1—O5—C23	−19.3 (4)
C1—Co1—C6—C5	88.57 (16)	O3—K1—O5—C23	−160.8 (4)
C8—Co1—C6—C5	−178.81 (16)	N1—K1—O5—C23	150.2 (5)
C3—Co1—C6—C5	−53.05 (18)	N2—K1—O5—C23	−30.8 (5)
C2—Co1—C6—C5	56.79 (18)	C24'—K1—O5—C23	−4.4 (4)
C4—Co1—C6—C5	−84.46 (16)	C22—O5—C23—C24	−174.9 (7)
C7—Co1—C6—C5	−176.99 (16)	K1—O5—C23—C24	50.0 (6)
C1—Co1—C7—C8	−85.10 (17)	O5—C23—C24—O6	−63.4 (6)
C5—Co1—C7—C8	179.58 (16)	C23—C24—O6—C25	179.7 (6)
C3—Co1—C7—C8	54.97 (19)	C23—C24—O6—K1	46.1 (5)
C2—Co1—C7—C8	−52.91 (19)	O5'—K1—O6—C25	−155.0 (4)
C4—Co1—C7—C8	87.38 (17)	O1—K1—O6—C25	131.8 (4)
C6—Co1—C7—C8	−177.86 (17)	O6'—K1—O6—C25	−81.1 (6)
C1—Co1—C8—C7	96.59 (17)	O2—K1—O6—C25	73.7 (4)
C5—Co1—C8—C7	−2.0 (8)	O5—K1—O6—C25	−144.7 (4)
C3—Co1—C8—C7	−133.72 (17)	O4—K1—O6—C25	−24.1 (4)
C2—Co1—C8—C7	136.70 (17)	O3—K1—O6—C25	−76.8 (4)
C4—Co1—C8—C7	−93.61 (17)	N1—K1—O6—C25	−155.5 (4)
C6—Co1—C8—C7	2.8 (2)	N2—K1—O6—C25	23.3 (4)
O5'—K1—N1—C15	141.1 (2)	C24'—K1—O6—C25	179.5 (5)
O1—K1—N1—C15	−105.27 (13)	O5'—K1—O6—C24	−25.0 (3)
O6'—K1—N1—C15	125.90 (14)	O1—K1—O6—C24	−98.2 (2)
O2—K1—N1—C15	−98.74 (12)	O6'—K1—O6—C24	48.8 (4)
O5—K1—N1—C15	131.10 (17)	O2—K1—O6—C24	−156.3 (2)
O4—K1—N1—C15	19.55 (13)	O5—K1—O6—C24	−14.7 (2)
O6—K1—N1—C15	141.63 (13)	O4—K1—O6—C24	105.9 (2)
O3—K1—N1—C15	9.80 (11)	O3—K1—O6—C24	53.2 (3)
N2—K1—N1—C15	84 (2)	N1—K1—O6—C24	−25.6 (3)
C24'—K1—N1—C15	149.41 (14)	N2—K1—O6—C24	153.3 (3)
O5'—K1—N1—C21'	17.4 (10)	C24'—K1—O6—C24	−50.5 (3)
O1—K1—N1—C21'	131.0 (10)	C24—O6—C25—C26	171.8 (7)
O6'—K1—N1—C21'	2.2 (10)	K1—O6—C25—C26	−55.4 (7)
O2—K1—N1—C21'	137.6 (10)	O6—C25—C26—N2	65.9 (15)
O5—K1—N1—C21'	7.4 (10)	C15—N1—C21'—C22'	−166.0 (11)
O4—K1—N1—C21'	−104.1 (10)	C9—N1—C21'—C22'	74.3 (15)
O6—K1—N1—C21'	17.9 (10)	K1—N1—C21'—C22'	−42.5 (17)
O3—K1—N1—C21'	−113.9 (10)	N1—C21'—C22'—O5'	52 (2)
N2—K1—N1—C21'	−39 (3)	C21'—C22'—O5'—C23'	168.0 (13)
C24'—K1—N1—C21'	25.7 (10)	C21'—C22'—O5'—K1	−36.6 (17)
O5'—K1—N1—C21	24.8 (7)	O1—K1—O5'—C22'	−42.3 (10)
O1—K1—N1—C21	138.4 (7)	O6'—K1—O5'—C22'	174.6 (10)
O6'—K1—N1—C21	9.6 (7)	O2—K1—O5'—C22'	−94.6 (10)
O2—K1—N1—C21	144.9 (7)	O5—K1—O5'—C22'	119 (2)
O5—K1—N1—C21	14.8 (7)	O4—K1—O5'—C22'	114.4 (10)
O4—K1—N1—C21	−96.8 (7)	O6—K1—O5'—C22'	−168.8 (10)
O6—K1—N1—C21	25.3 (7)	O3—K1—O5'—C22'	53.1 (10)
O3—K1—N1—C21	−106.5 (7)	N1—K1—O5'—C22'	10.7 (10)
N2—K1—N1—C21	−32 (2)	N2—K1—O5'—C22'	−170.4 (10)
C24'—K1—N1—C21	33.1 (7)	C24'—K1—O5'—C22'	−157.9 (10)
O5'—K1—N1—C9	−97.22 (19)	O1—K1—O5'—C23'	112.6 (6)
O1—K1—N1—C9	16.42 (11)	O6'—K1—O5'—C23'	−30.5 (6)
O6'—K1—N1—C9	−112.41 (14)	O2—K1—O5'—C23'	60.2 (7)
O2—K1—N1—C9	22.94 (13)	O5—K1—O5'—C23'	−86 (2)
O5—K1—N1—C9	−107.22 (17)	O4—K1—O5'—C23'	−90.8 (7)
O4—K1—N1—C9	141.23 (11)	O6—K1—O5'—C23'	−13.9 (6)
O6—K1—N1—C9	−96.68 (13)	O3—K1—O5'—C23'	−152.1 (6)
O3—K1—N1—C9	131.49 (13)	N1—K1—O5'—C23'	165.6 (7)
N2—K1—N1—C9	−154 (2)	N2—K1—O5'—C23'	−15.5 (7)
C24'—K1—N1—C9	−88.90 (14)	C24'—K1—O5'—C23'	−3.1 (6)
C15—N1—C9—C10	74.3 (2)	C22'—O5'—C23'—C24'	163.2 (10)
C21'—N1—C9—C10	−166.1 (8)	K1—O5'—C23'—C24'	5.2 (10)
C21—N1—C9—C10	−163.9 (6)	O5'—C23'—C24'—O6'	50.6 (9)
K1—N1—C9—C10	−47.21 (18)	O5'—C23'—C24'—K1	−3.9 (8)
N1—C9—C10—O1	62.7 (2)	O5'—K1—C24'—O6'	−111.7 (3)
C9—C10—O1—C11	170.04 (16)	O1—K1—C24'—O6'	175.0 (2)
C9—C10—O1—K1	−44.9 (2)	O2—K1—C24'—O6'	113.3 (2)
O5'—K1—O1—C10	67.5 (2)	O5—K1—C24'—O6'	−98.6 (3)
O6'—K1—O1—C10	113.59 (17)	O4—K1—C24'—O6'	10.4 (3)
O2—K1—O1—C10	−157.85 (15)	O6—K1—C24'—O6'	36.3 (3)
O5—K1—O1—C10	64.40 (19)	O3—K1—C24'—O6'	−61.1 (3)
O4—K1—O1—C10	−88.23 (14)	N1—K1—C24'—O6'	−121.7 (2)
O6—K1—O1—C10	124.81 (15)	N2—K1—C24'—O6'	57.2 (2)
O3—K1—O1—C10	−36.68 (14)	O5'—K1—C24'—C23'	2.6 (5)
N1—K1—O1—C10	15.61 (13)	O1—K1—C24'—C23'	−70.7 (5)
N2—K1—O1—C10	−164.61 (13)	O6'—K1—C24'—C23'	114.3 (6)
C24'—K1—O1—C10	110.63 (16)	O2—K1—C24'—C23'	−132.4 (5)
O5'—K1—O1—C11	−148.9 (2)	O5—K1—C24'—C23'	15.7 (5)
O6'—K1—O1—C11	−102.79 (16)	O4—K1—C24'—C23'	124.7 (5)
O2—K1—O1—C11	−14.23 (12)	O6—K1—C24'—C23'	150.6 (7)
O5—K1—O1—C11	−151.98 (18)	O3—K1—C24'—C23'	53.2 (5)
O4—K1—O1—C11	55.39 (14)	N1—K1—C24'—C23'	−7.4 (5)
O6—K1—O1—C11	−91.56 (14)	N2—K1—C24'—C23'	171.6 (5)
O3—K1—O1—C11	106.94 (12)	C23'—C24'—O6'—C25'	162.5 (9)
N1—K1—O1—C11	159.24 (14)	K1—C24'—O6'—C25'	−123.5 (8)
N2—K1—O1—C11	−20.99 (14)	C23'—C24'—O6'—K1	−74.0 (6)
C24'—K1—O1—C11	−105.75 (15)	O5'—K1—O6'—C24'	51.1 (3)
C10—O1—C11—C12	−168.94 (17)	O1—K1—O6'—C24'	−6.6 (3)
K1—O1—C11—C12	44.87 (19)	O2—K1—O6'—C24'	−73.3 (3)
O1—C11—C12—O2	−63.1 (2)	O5—K1—O6'—C24'	60.7 (3)
C11—C12—O2—C13	−179.82 (17)	O4—K1—O6'—C24'	−170.6 (2)
C11—C12—O2—K1	50.57 (19)	O6—K1—O6'—C24'	−46.7 (4)
O5'—K1—O2—C12	46.6 (2)	O3—K1—O6'—C24'	136.8 (2)
O1—K1—O2—C12	−19.64 (12)	N1—K1—O6'—C24'	66.4 (3)
O6'—K1—O2—C12	111.45 (14)	N2—K1—O6'—C24'	−114.4 (3)
O5—K1—O2—C12	54.0 (2)	O5'—K1—O6'—C25'	174.7 (6)
O4—K1—O2—C12	−155.39 (12)	O1—K1—O6'—C25'	117.0 (5)
O6—K1—O2—C12	104.87 (13)	O2—K1—O6'—C25'	50.3 (5)
O3—K1—O2—C12	−97.02 (12)	O5—K1—O6'—C25'	−175.7 (5)
N1—K1—O2—C12	−26.21 (13)	O4—K1—O6'—C25'	−47.0 (5)
N2—K1—O2—C12	153.72 (13)	O6—K1—O6'—C25'	76.9 (6)
C24'—K1—O2—C12	87.09 (15)	O3—K1—O6'—C25'	−99.6 (5)
O5'—K1—O2—C13	−81.8 (2)	N1—K1—O6'—C25'	−170.0 (5)
O1—K1—O2—C13	−147.97 (13)	N2—K1—O6'—C25'	9.1 (5)
O6'—K1—O2—C13	−16.88 (15)	C24'—K1—O6'—C25'	123.6 (6)
O5—K1—O2—C13	−74.3 (2)	C24'—O6'—C25'—C26'	76.4 (14)
O4—K1—O2—C13	76.27 (13)	K1—O6'—C25'—C26'	−36.8 (13)
O6—K1—O2—C13	−23.46 (14)	O6'—C25'—C26'—N2	62 (2)
O3—K1—O2—C13	134.64 (12)	C13—C14—N2—C20	−160.38 (17)
N1—K1—O2—C13	−154.55 (12)	C13—C14—N2—C26	80.2 (8)
N2—K1—O2—C13	25.38 (12)	C13—C14—N2—C26'	77.3 (12)
C24'—K1—O2—C13	−41.25 (15)	C13—C14—N2—K1	−40.0 (2)
C12—O2—C13—C14	172.92 (17)	C19—C20—N2—C14	77.9 (2)
K1—O2—C13—C14	−57.19 (19)	C19—C20—N2—C26	−166.5 (5)
O2—C13—C14—N2	67.6 (2)	C19—C20—N2—C26'	−154.5 (6)
C21'—N1—C15—C16	82.6 (9)	C19—C20—N2—K1	−41.77 (19)
C21—N1—C15—C16	73.3 (7)	C25—C26—N2—C14	−161.0 (10)
C9—N1—C15—C16	−161.52 (17)	C25—C26—N2—C20	80.2 (13)
K1—N1—C15—C16	−41.33 (19)	C25—C26—N2—K1	−43.2 (15)
N1—C15—C16—O3	64.8 (2)	C25'—C26'—N2—C14	−166.7 (14)
C15—C16—O3—C17	172.92 (15)	C25'—C26'—N2—C20	70 (2)
C15—C16—O3—K1	−52.83 (18)	C25'—C26'—N2—K1	−47 (2)
O5'—K1—O3—C17	113.19 (17)	O5'—K1—N2—C14	128.55 (19)
O1—K1—O3—C17	−152.93 (12)	O1—K1—N2—C14	14.46 (14)
O6'—K1—O3—C17	52.14 (15)	O6'—K1—N2—C14	142.81 (15)
O2—K1—O3—C17	−95.13 (12)	O2—K1—N2—C14	7.78 (11)
O5—K1—O3—C17	104.59 (15)	O5—K1—N2—C14	138.41 (16)
O4—K1—O3—C17	−15.30 (11)	O4—K1—N2—C14	−109.89 (13)
O6—K1—O3—C17	50.80 (16)	O6—K1—N2—C14	126.87 (14)
N1—K1—O3—C17	154.78 (13)	O3—K1—N2—C14	−101.34 (12)
N2—K1—O3—C17	−23.96 (13)	N1—K1—N2—C14	−175 (25)
C24'—K1—O3—C17	78.64 (18)	C24'—K1—N2—C14	119.41 (15)
O5'—K1—O3—C16	−18.86 (18)	O5'—K1—N2—C20	−110.98 (19)
O1—K1—O3—C16	75.01 (12)	O1—K1—N2—C20	134.93 (11)
O6'—K1—O3—C16	−79.91 (15)	O6'—K1—N2—C20	−96.72 (14)
O2—K1—O3—C16	132.81 (11)	O2—K1—N2—C20	128.26 (13)
O5—K1—O3—C16	−27.46 (15)	O5—K1—N2—C20	−101.12 (16)
O4—K1—O3—C16	−147.35 (13)	O4—K1—N2—C20	10.59 (11)
O6—K1—O3—C16	−81.25 (15)	O6—K1—N2—C20	−112.65 (14)
N1—K1—O3—C16	22.73 (11)	O3—K1—N2—C20	19.13 (13)
N2—K1—O3—C16	−156.01 (11)	N1—K1—N2—C20	−55 (2)
C24'—K1—O3—C16	−53.42 (18)	C24'—K1—N2—C20	−120.12 (15)
C16—O3—C17—C18	179.74 (15)	O5'—K1—N2—C26	13.0 (8)
K1—O3—C17—C18	45.90 (17)	O1—K1—N2—C26	−101.1 (8)
O3—C17—C18—O4	−63.4 (2)	O6'—K1—N2—C26	27.3 (8)
C17—C18—O4—C19	−177.62 (16)	O2—K1—N2—C26	−107.7 (8)
C17—C18—O4—K1	49.20 (18)	O5—K1—N2—C26	22.9 (8)
O5'—K1—O4—C19	128.3 (3)	O4—K1—N2—C26	134.6 (8)
O1—K1—O4—C19	−84.10 (12)	O6—K1—N2—C26	11.4 (8)
O6'—K1—O4—C19	80.62 (14)	O3—K1—N2—C26	143.1 (8)
O2—K1—O4—C19	−29.43 (12)	N1—K1—N2—C26	69 (2)
O5—K1—O4—C19	127.49 (19)	C24'—K1—N2—C26	3.9 (8)
O6—K1—O4—C19	68.32 (13)	O5'—K1—N2—C26'	3.7 (10)
O3—K1—O4—C19	−149.41 (13)	O1—K1—N2—C26'	−110.4 (10)
N1—K1—O4—C19	−159.29 (11)	O6'—K1—N2—C26'	18.0 (10)
N2—K1—O4—C19	21.90 (11)	O2—K1—N2—C26'	−117.1 (10)
C24'—K1—O4—C19	76.26 (15)	O5—K1—N2—C26'	13.6 (10)
O5'—K1—O4—C18	−100.7 (3)	O4—K1—N2—C26'	125.3 (10)
O1—K1—O4—C18	46.94 (13)	O6—K1—N2—C26'	2.0 (10)
O6'—K1—O4—C18	−148.34 (14)	O3—K1—N2—C26'	133.8 (10)
O2—K1—O4—C18	101.61 (12)	N1—K1—N2—C26'	60 (3)
O5—K1—O4—C18	−101.47 (19)	C24'—K1—N2—C26'	−5.4 (10)

### Hydrogen-bond geometry (Å, º)

**Table:**

*D*—H···*A*	*D*—H	H···*A*	*D*···*A*	*D*—H···*A*
C24—H24*A*···O5^i^	0.99	2.59	3.326 (6)	131

Symmetry code: (i) −*x*+1, *y*, −*z*+3/2.
